# Alternative Splicing Regulation of Cancer-Related Pathways in *Caenorhabditis elegans*: An In Vivo Model System with a Powerful Reverse Genetics Toolbox

**DOI:** 10.1155/2013/636050

**Published:** 2013-08-28

**Authors:** Sergio Barberán-Soler, James Matthew Ragle

**Affiliations:** ^1^SomaGenics, Inc., Santa Cruz, CA 95060, USA; ^2^Department of Molecular, Cell and Developmental Biology, The Center for Molecular Biology of RNA, University of California, Santa Cruz, Santa Cruz, CA 95064, USA

## Abstract

Alternative splicing allows for the generation of protein diversity and fine-tunes gene expression. Several model systems have been used for the in vivo study of alternative splicing. Here we review the use of the nematode *Caenorhabditis elegans* to study splicing regulation in vivo. Recent studies have shown that close to 25% of genes in the worm genome undergo alternative splicing. A big proportion of these events are functional, conserved, and under strict regulation either across development or other conditions. Several techniques like genome-wide RNAi screens and bichromatic reporters are available for the study of alternative splicing in worms. In this review, we focus, first, on the main studies that have been performed to dissect alternative splicing in this system and later on examples from genes that have human homologs that are implicated in cancer. The significant advancement towards understanding the regulation of alternative splicing and cancer that the *C. elegans* system has offered is discussed.

## 1. Introduction

Since the early 1960s with the efforts of Sydney Brenner, the nematode *Caenorhabditis elegans* has been established as a popular model organism in developmental biology and neurobiology. There are many biological advantages that make it an attractive system for several fields of research. The adult worm contains 959 somatic cells, making its anatomy relatively simple. Experiments in the late 1970s showed that it has an invariant cell lineage during establishment of the somatic tissues [1]. It has two sexes, a self-fertilizing hermaphrodite and males, allowing genetic crosses to be performed. *C. elegans* has a short life cycle of less than three days and each hermaphrodite produces about 300 progeny by self-fertilization or up to 1000 progeny from cross progeny with males. The gonad is a relatively large organ in this animal, allowing for studies of organogenesis, cell proliferation, meiosis, and embryogenesis. During its development, the hermaphrodite worms produce sperm at one stage of their life cycle before switching to produce oocytes. The molecular pathways of this sperm to oocyte transition have been studied extensively [2]. Its complete genome, sequenced in 1998, was the first sequenced genome from a multicellular organism. It has a genome size of 97 megabases containing close to 19,000 protein coding genes [3]. Genome-wide alignments with other five related nematodes are now available for any comparative genomics approach [4]. The modENCODE project systematically generated genome-wide data from transcriptome profiling, transcription factor-binging sites, and maps of chromatin organization to improve genome annotation [5]. When comparing the *C. elegans* genome to higher eukaryotes, it was found that close to 40% of the genes that have been associated with diseases in humans have worm orthologues, and cancer is not the exception [6].

## 2. Alternative Splicing Prevalence

**Figure 1:**
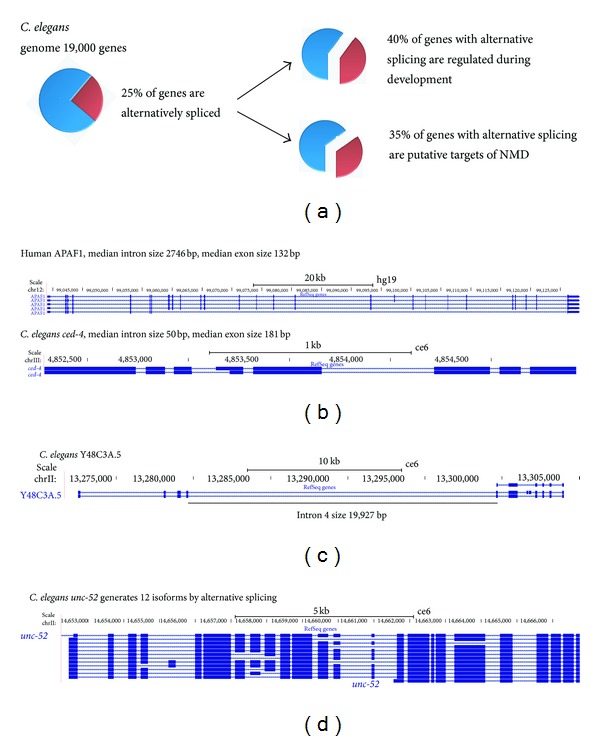
*Caenorhabditis elegans* alternative splicing events. (a) Genome-wide analysis of alternative splicing in *C. elegans*; (b) comparison of the human APAF1 and the *C. elegans* homolog *ced-4* gene models shows significant differences in intron size between species for genes with important alternative splicing events; (c) Y48C3A.5 intron 4 (19,927 bp) is one example of the 144 introns in the *C. elegans* genome that are more than 10 kb in length; (d) *C. elegans unc-52* gene undergoes complex alternative splicing that generates at least 12 different isoforms by the use of nine different cassette exons.

In 1990, the first report of an alternative splicing event in the *C. elegans* genome was published. Interestingly the event corresponds to the PKA mRNA, a kinase implicated in the onset and progression of several cancers [7]. Since then, several groups have used different approaches to predict the percentage of genes in the *C. elegans* genome that undergo alternative splicing. Initial estimates using a limited number of Expressed Sequence Tags (ESTs) predicted fewer than 1000 genes to be alternatively spliced [8]. By normalizing the occurrence of alternative splicing, taking into account the coverage of ESTs, it was estimated that close to 10% of the *C. elegans* genome undergoes alternative splicing [9]. More recent analysis using next-generation sequencing identified 8,651 putative novel splicing events, suggesting that up to 25% of genes have an alternative splicing event (Figure 1(a)) [10]. While this percentage is far from the >90% reported for the human genome, it does show that alternative splicing in *C. elegans* is not an uncommon mechanism to generate protein diversity.

## 3. Effects of Genome Compaction on AS

The worm genome appears to be under selective pressure to promote a reduction in genome size [11]. This natural selection towards a small genome can be seen in features like short intergenic regions, short UTRs, and small introns [8, 12, 13]. For example, in humans, the median size of introns in the coding sequences is 1,334 bases, while in worms the average intron is just 65 bases (Figure 1(b)) [4, 14]. More than 20 years ago, the small size of *C. elegans* introns was already the subject of study. It was demonstrated that a short 53 nucleotide worm intron could not be efficiently spliced in human extracts, while an expansion of this intron with 31 extra nucleotides allowed for efficient splicing [15]. In spite of this reduction in intron size, the worm spliceosome is still capable of removing big introns (144 introns in the *C. elegans* genome are bigger than 10 kb (Figure 1(c)) [4]). Larger intron size has been correlated with alternative splicing in several species [16]. Worms also have complex patterns of alternative splicing where multiple exons in the same gene are alternatively spliced to generate multiple isoforms (Figure 1(d)). This means that the information content of a worm intron is on average greater than in higher eukaryotes (higher density of functional elements in introns). This makes the molecular dissection of worm introns easier to achieve. By using genomic alignments between two *Caenorhabditis* species, the identification of novel intronic elements important for alternative splicing regulation has been described [17]. Other groups have also used comparative genomics together with UV cross-linking and Electrophoretic Mobility Shift Assays (EMSA) to identify cis-elements important for alternative splicing regulation [18].

## 4. Evidence That AS in Nematodes Is under Stabilizing Selection

Several studies comparing alternative splicing events in mammals and insects concluded that a high percentage of the events are not conserved and are species specific [19, 20]. This high variability in alternative splicing makes it necessary to validate the functionality of the events studied. To test whether the smaller percentage of genes with alternative splicing in worms also follows these patterns of high variability during evolution, several groups have measured the levels of conservation between different *C. elegans* populations or between related species [21, 22]. In comparison to the findings in higher eukaryotes, the regulation of alternative splicing in natural populations of nematodes appears to be under strong stabilizing selection with low intra- an interspecies variability. These results point to an essential intrinsic characteristic of alternative splicing in worms: its functionality. The detection of alternative splicing in a *C. elegans* transcript has a higher probability of being a functional and regulated event than in other systems.

## 5. Alternative Splicing Regulation

A proxy for the functionality of an alternative splicing event that has been used for other systems is its regulation. If a particular event is detected as regulated across different conditions or during development then the possibility that the isoforms have specific functions is higher. In the last five years *C. elegans* joined other species in terms of the detection of changes in alternative splicing at genome-wide levels. Initially with the use of splicing-sensitive microarrays and later with the use of next-generation sequencing the regulation of alternative splicing in worms has been studied in detail across different conditions and mutations [10, 23, 24]. Initial measurement of changes in splicing during development demonstrated that up to 40% of the events detected are regulated (>2 fold) between different stages of worms (Figure 1(a)) [23]. This result was further validated with tiling arrays and next-generation sequencing [24]. Several examples of tissue-specific splicing are known. The alternative splicing regulation of a neuron-specific exon of *unc-32*, the worm *a* subunit of V_0_ complex of vacuolar-type H^+^-ATPases, was recently characterized [25]. A male-specific isoform of *unc-55*, a transcription factor, has also been reported [26]. While individual examples of tissue-specific splicing are known, a genome-wide analysis of tissue-specific splicing is still missing. With the increased sensitivity of next-generation sequencing techniques, together with the availability of manual or molecular dissections that allow the isolation of tissue-specific mRNA (mRNA-tagging [27]) a complete catalog of tissue-specific splicing should be possible in the near future. 

While a big set of splicing events produce two protein isoforms, another important group introduces premature termination codons (PTC) to one of the isoforms. It is known that the introduction of a PTC to one isoform targets it to nonsense-mediated decay (NMD) (reviewed in [28]). NMD mutants were first described in *C. elegans* almost 25 years ago [29]. Contrary to other systems where mutations in NMD factors are lethal, in worms, null mutations for all the seven core factors of NMD are viable (*smg-1* to *smg-7*). This has facilitated the study of AS coupled to NMD in worms. Some of the first events of wild-type transcripts that were shown to be regulated by NMD are splicing factors in the *C. elegans* genome [30]. An important discovery concerning the regulation of alternative splicing in worms is that between 20–35% of the events in the genome appear to be targets of NMD (Figure 1(a)) [24, 31]. The conclusion from these studies is, then, that alternative splicing in *C. elegans* is not just a generator of protein diversity but also an important regulator that fine-tunes gene expression levels by targeting specific isoforms for degradation. Furthermore, it has been proposed that NMD in worms can also be regulated with the potential to stabilize particular NMD targets allowing them to be translated into truncated proteins with putative dominant negative functions [31].

## 6. Powerful Reverse Genetics to Dissect Alternative Splicing

One of the advantages of *C. elegans* as a model system is the availability of powerful tools for reverse genetics. Any laboratory can obtain stable mutants for many genes from the Caenorhabditis Genetics Center (CGC) at The University of Minnesota. The National Bioresource Project in Japan runs a program where mutants for a gene of interest can be requested and they are obtained at the facility by a protocol involving random mutagenesis with TMP/UV. These two centers allow for any group to obtain mutant strains for the gene of interest in an inexpensive and expedited manner. Recently, a more ambitious project to create a million different mutants has been performed by the Moerman and Waterston labs [32]. The aim of this project was to use next-generation sequencing and a collection of 2000 mutagenized strains to identify multiple mutations in virtually all the genes in the worm genome. This project will likely allow the study of mutant worms that have mutations in isoform-specific regions. All these resources allow for the use of stable mutants to characterize the roles of either putative splicing factors or more specifically of particular isoforms. This approach to screen the effects that mutations in different splicing factors have on a particular splicing event has been used before [33]. The conclusion from this work was that the coregulation of alternative splicing by diverse factors is a common phenomenon in worms.

Another great resource for the worm community is the availability of genome-wide RNAi libraries [34]. Researchers aiming to characterize genetic pathways have used these libraries for genome-wide screens. Some of them have found that several splicing-related components have interesting phenotypes when targeted by RNAi [35]. More focused screens with a subset of clones from these libraries are used to characterize a specific group of genes. For example, Kerins et al. used an RNAi screen of all the predicted *C. elegans* splicing factors and found that many of them showed an overproliferation phenotype in a sensitized germline background [36]. 

## 7. In Vivo Alternative Splicing Reporters

One of the most advantageous tools for the study of alternative splicing in *C. elegans* is the use of bichromatic alternative splicing reporters [37]. This transgenic reporter system allows the visualization of splicing events by tagging each one of the isoforms with a different fluorescent protein. A worm population with differences in splicing regulation can then be sorted using FACS cytometry and worms with a particular splicing ratio obtained. This together with the use of mutagenesis or RNAi allows performing an in vivo screen for regulators of any splicing event of interest. The advantage of these reporters in worms is that the characterization of an alternative splicing event can be performed in vivo. Different events that have been studied with this technology are *egl-15*, *let-2*, *unc-60*, and *unc-32* [25, 37–40]. This technology has allowed the identification of splicing factors that regulate these events, as well as the cis-elements that are necessary for its regulation.

## 8. *C. elegans* AS Regulation of Cancer-Related Pathways

**Figure 2:**
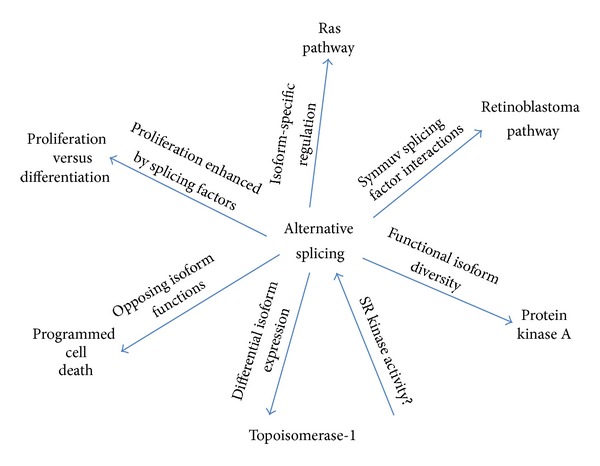
Connections between alternative splicing in *C. elegans* and pathways homologous to those that cause excessive cell proliferation or apoptosis in humans, as reviewed in this paper.

Given the diversity of tools available to perform studies in *C. elegans*, researchers have set out to understand details about cancer that were long a mystery. Questions surrounding cell proliferation and cell-cell communication as well as the cellular and molecular components that make up a stem cell niche often must be answered in a multicellular context. Furthermore, the fact that biological processes and factors active in splicing and human cancers are almost wholly homologous with those in *C. elegans* makes the move to worm studies rewarding. Disrupted cancer pathways in *C. elegans*, for the most part, have clear, simple, and observable phenotypes that extend beyond just cell death or proliferation. Using these pathways as tools and read-outs, splicing factors and splicing events have been identified, in *C. elegans*, to interact with, regulate, and cooperate with homologous pathways that lead to cancer in humans (Figure 2).

### 8.1. Extensive Splicing of Ras/*let-60* Pathway Components Contributes to Cell Fate Determination and Proliferation

Overwhelming evidence links cell signaling and gene expression changes in the promotion of human cancers. Alternative splicing plays a major role in defining the activity of signal transduction pathways such as Ras/*let-60*. Strikingly, of the 50–60 genes that are known components of, regulate or, are directly regulated by the Ras/*let-60 *pathway in *C. elegans* [41], approximately half of them produce multiple isoforms differing by at least one alternative exon [4]. This highlights the importance of proper gene expression through post-transcriptional regulation (Table 1).

The ligand-dependent Ras pathway in *C. elegans* stimulates the induction of vulval precursor cells (VPCs) in the hypodermis to divide and differentiate into a functional vulva during larval development [42, 43]. Increased signaling from the Ras pathway leads to a multivulval (Muv) phenotype, while decreased signaling causes a vulvaless (Vul) phenotype [44]. These simple and observable phenotypes provide an excellent system to ascertain the contributions of alternative splicing to Ras signaling activity. The *C. elegans* homolog of the Ras-MAPK pathway stimulant Epidermal Growth Factor, *lin-3*, produces several isoforms with unique characteristics. LIN-3 isoform specialization of function has previously been observed in pathways aside from Ras-mediated cell signaling and has been found to differentially mediate growth rates, feeding behavior, and cellular quiescence [45]. In VPCs, the activity of the LIN-3L isoform is dependent on interaction with the *C. elegans* Rhomboid protease homolog, ROM-1, while the activity of LIN-3S is not [46]. LIN-3S is expressed in a specialized Anchor Cell (AC) responsible for inducing a primary set of VPCs during larval development, and LIN-3L expression and possible secretion by primary VPCs themselves act as a ligand stimulant of the Ras pathway in secondary VPCs [42, 43]. This stimulation may cause the secondary VPCs to switch their cell fate from hypodermis to vulva [43]. It is unknown if coordinated expression of these isoforms from distinct cell types establishes a gradient through Ras signaling that induces nearby VPCs to proceed through necessary cell divisions to develop into a proper vulva.

RasGAPs are GTPase-activating proteins that specifically promote the hydrolysis of Ras-bound GTP molecules, thereby inactivating the Ras molecule and its signaling [47]. *gap-2*, in *C. elegans*, is similar to the p120 Ras-GAP family [48]. The gene contains 25 exons that use alternative splicing and transcription start sites to produce 9 mRNA products [49]. These isoforms were identified by a rapid amplification of cDNA end (5′RACE) technique using primers specific to a sequence common to many mature mRNAs in *C. elegans*, the SL1 trans-splice leader. This 22nt sequence is naturally spliced onto 5′ ends of transcripts from ~70% of genes [50]. In this case, they served as 5′ primer binding sites in reverse transcription reactions followed by PCR to identify isoforms with alternative 5′ ends and promoters [46]. Differential expression of *gap-2* isoforms in several tissue types was revealed by expression of transgenes containing these alternative *gap-2* promoters fused to GFP in *C. elegans.* The function of RasGAP proteins from various genes in other organisms [51] may be relegated to multiple isoforms from a small number of RasGAP genes producing many isoforms in *C. elegans*. While the effects of this dynamic expression pattern of isoforms on molecular signaling pathways such as the Ras pathway are difficult to tease apart, target sites for PKC, PKA, and protein tyrosine kinases present or absent in each isoform [49] may provide clues to individual isoform activities. 

### 8.2. Redundant Retinoblastoma/*lin-35* Pathways Are Populated by Splicing Factors

Homozygous mutations of the retinoblastoma (Rb) gene have been shown to promote retinal cancer, small-cell lung carcinomas, and osteosarcomas [52]. In worms, a single homolog of Rb, *lin-35*, represses the multivulval phenotype observed in Ras pathway hyper-signaling mutants [53]. Two redundant pathways, termed synthetic multivulval (synmuv) class A and class B, are active in the VPCs and induce changes in expression of genes that lead to a multivulval fate through histone modification and chromatin remodeling [53, 54]. To induce a phenotype, disruptive mutations in single genes within each class must be present. Interestingly, mutations in the *lin-15* locus created alleles with Muv, class A synmuv, and class B synmuv phenotypes, leading to speculation that two genes existed at the locus with distinct functions in each pathway. In RNAi screens for interactors of the class B synmuv pathway in *C. elegans*, 57 genes were found to enhance the Muv phenotype observed in *lin-35* or other synmuv class B mutant lines [35, 55]. Ten of these potential interactors (*rsr-2*, *lsm-2/gut-2*, *lsm-4*, *snr-1–7*) have roles in splicing. Their synmuv B mutant phenotypes are dependent on Ras activity and *lin-3* ligand binding. While the activities, interactions, and mutant phenotypes of most of these splicing-related Rb enhancers are not well understood, RNAi of some of the snr genes (*snr-1, snr-2, snr-4, snr-5, snr-6*, and *snr-7*) led to embryonic lethality and nuclear pore organization disruption [56]. The question of whether these phenotypes arise from disrupted functions that have no role in splicing regulation is still unanswered, but evidence is mounting supporting the idea that constitutive splicing and alternative splicing are major contributors in *C. elegans* to pathways that, when altered, lead to cancer in human tissues. Undoubtedly, future studies in *C. elegans* will enrich current knowledge concerning the interwoven activities of splicing and the Rb/*lin-35* pathway.

### 8.3. Protein Kinase A Isoform Diversity Replaces the Need for Multiple Genetic Loci

Protein kinase A (PK-A) is involved in many cellular processes and has been implicated in several cancers [57]. The mammalian PK-A is composed of two distinct subunits, the regulatory and catalytic, that are each interchangeable with protein subunits from several genetic loci. The two subunits of PK-A are conserved in *C. elegans* [7] but derived from possibly only three genes, *kin-1*, *kin-2*, and F47F2.1. Interestingly, the modularity of the subunits that make up PK-A in *C. elegans* has been conserved through alternative usage of exons. The catalytic subunit (C-subunit) alone is thought to undergo alternative splicing at both N- and C-termini to create at least 12 different isoforms [58]. These isoforms are conserved in *C. briggsae*, a relative of *C. elegans*, and show differential expression during development, suggesting that the diversity of potential subunits itself has functional importance [59]. An isoform of the C-subunit containing the N′1 exon harbors a myristoylation site and is highly expressed in eggs, where an alternative exon N′4 lacking the myristoylation site is highly expressed in adult worms [59]. The presence of N′1 protein isoform expression and myristoylation was found to affect substrate targeting of PK-A but not the catalytic activity of the enzyme itself [60]. Similarly, isoforms of the regulatory (R-subunit) have recently been identified and found to contain or lack domains necessary for docking to A-kinase anchor proteins and the C-subunit of the PK-A holoenzyme [61]. A second gene on the X chromosome of *C. elegans*, F47F2.1, has homology to murine C*α* subunits [58]. Like the kin-1 N′4 isoform, expression of the longer F47F2.1b isoform is low in eggs but high in mixed populations of worms [59]. A truncated isoform, F47F2.1a, lacks amino acids important for ATP-binding, and knockdown of N′4 *kin-1* isoform has no obvious phenotype leading to speculation that some of these isoforms may be redundant or unnecessary [59, 62]. 

### 8.4. Topoisomerase-1 Isoforms Have Differential Expression and Potential SR Kinase Activity

Isoforms from the same genetic locus often have distinct expression patterns and functions that are seemingly unrelated. *C.elegans* DNA topoisomerase I (*top-1*) produces two isoforms that vary in temporal and spatial expression patterns. Cellular and subcellular localization differences between the isoforms lead to speculation they may function in different processes. The TOP-1*β* isoform skips exon 2 and is more ubiquitously expressed throughout *C. elegans* development, being found in multiple cell types and all stages from embryo through adulthood. Conversely, the inclusion isoform, TOP-1*α*, is detectably present in embryos and, interestingly, in neurons at the comma stage, then decreases in abundance as worms enter larval stages [63]. Isoform-specific immuno-histochemical localization assays in germline cells identified TOP-1*β* concentration in nucleoli and TOP-1*α* concentration at centrosomes and on chromosomes [64, 65]. Expression of a GFP transgene driven by the topoisomerase promoter was also detected strongly in the distal tip cell of L3/L4 worms indicating that somatic gonad sheath development and/or germline stem cell proliferation may be regulated by TOP-1 activity. RNAi of *C. elegans top-1* reduced the number of germ cells in the mature germline by anywhere from 50–100% [65]. It is currently unknown how *top-1* isoforms contribute to the maintenance of stem cell proliferation or exactly which isoform would be performing such functions. Localization of the inclusion isoform, TOP-1*α*, to centrosomes and chromosomes suggests it could be involved in chromosome segregation, in regulation of transcription, or possibly in posttranscriptional regulation. DNA topoisomerase I was identified as an SR protein kinase in HeLa cell extracts [66]. It was found to phosphorylate splicing factors involved in cell cycle regulation, such as SF2/ASF [67, 68]. It may be possible that these isoforms are working more closely than originally thought if one or both of the *top-1* isoforms affect the cell cycle and carry the same SR kinase activity as shown in HeLa cells. The activity of one or both of the TOP-1 isoforms as SR protein kinases suggests that *top-1* alternative splicing may be autoregulated. Kinase activity of TOP-1 and its autoregulation have yet to be determined in worms.

### 8.5. Splicing Factors as Regulators of Cell Proliferation and Differentiation

Several genes involved in splicing regulation have been implicated in the proliferation/differentiation decision in the *C. elegans* germline. *glp-1*(ar202gf) worms were used to perform an RNAi screen searching for genes that led to increased cell proliferation in the germline. The resulting genes included factors involved in every step of the splicing process (spliceosome construction initiation, reorganization of the snRNP complex and removal of the lariat; [36]). *prp-17*, for example, encodes an ortholog of yeast CDC40 and human prp17 and is involved in the 2nd catalytic step of intron removal in the spliceosome [36, 69]. RNAi of *prp-17* in the *rrf-1*(pk1417);*glp-1*(oz264) background enhanced germline tumors, suggesting that it is involved in the proliferation versus differentiation decision. Similarly, the mog genes represent *C. elegans* homologs of core yeast splicing factors that regulate the pathway downstream of *glp-1* to promote differentiation and the oocyte fate of maturing germ cells [70–72]. The U2 snRNP-associated splicing complex SF3b is essential for splicing [73, 74]. TEG-4 is the worm homolog of human SF3b subunit 3 (aka SAP130) that increases excessive cell proliferation in the *glp-1*(ar202gf) background [75]. Interestingly, epistasis experiments attempting to determine the genetic relationship between *teg-4* and the *glp-1*/Notch signaling pathway in various cell types were inconclusive. Human CD2BP2 was suggested to regulate splicing via U4_U5_U6 tri-snRNP formation [76]. TEG-1, the *C. elegans* homolog of CD2BP2 has been found to bind UAF-1, the worm U2AF large subunit homolog, suggesting that it could work in two important steps in the mechanism of pre-mRNA splicing [77]. *teg-1*(oz230), in combination with a *glp-1* mild gain-of-function mutant background, produces a tumorous germline phenotype as well. To what extent splicing factors, themselves, have individual genetic interactions within the proliferation versus differentiation pathways or if global splicing regulation is more indirectly influential is still unknown.

### 8.6. CED-4 Isoforms Promote and Inhibit Systematic Apoptosis during Development

Disruption of alternative splicing patterns may affect the onset of programmed cell death so intricately regulated in *C. elegans*. The worm homolog of APAF1, *ced-4*, was first identified as a core factor in the developmental induction of neuronal programmed cell death [78]. *ced-4* physically links *ced-3*, a member of a caspase family of proteases to *ced-9*, a cell death suppressor [79–81]. As a regulatory switch, *ced-4* is a core determinant of the decision of a cell to be systematically culled or not. *ced-4* encodes two protein isoforms, CED-4L and CED-4S, differing by 24 amino acids at the 5′ end of exon 4. While CED-4S normally promotes programmed cell death, overexpression of the CED-4L isoform led to ectopic cell growth and rescue of lethal phenotypes seen in *ced-9* loss-of-function mutants [82, 83]. It is CED-4L association with CED-3 that surprisingly inhibits cell death in a dominant negative manner. The presence of the longer P-loop in the long isoform still allows for association of CED-4L with the protease domain of CED-3 but disrupts the association of CED-4L with the prodomain of CED-3, which normally contributes to its activation [84]. Studies in mammalian cells have similarly identified *ced-3* and *ced-4* homologous genes that produce isoforms with opposing cell death functions [85, 86]. An imbalance in the concentrations of these isoforms or in the abundance or activities of specific SR or hnRNP proteins often leads to one developmental cell fate over the other [87]. Complex regulation of alternative splicing factors, therefore, may direct developmental apoptosis programs. Loss-of-function of an SR protein kinase, *spk-1*, in a *ced-4* partial loss-of-function background, as well as mutant alleles of several SR proteins themselves, leads to an increase in apoptosis [88], suggesting that control of alternative splicing factor activity may play a direct or indirect role in the regulation of developmentally programmed apoptosis. In vitro studies have shown that SPK-1 can bind and phosphorylate alternative splicing factors like SF2 and RNAi-mediated knockdown of *spk-1* in *C. elegans* causes embryonic lethality and germline development defects [89, 90]. Experiments aimed at understanding the complex code that dictates what genetic material is included or skipped in mature mRNA and subsequent protein products will undoubtedly clarify further the impact that alternative splicing has in the mediation of programmed cell death through factors such as *ced-4*.

### 8.7. Research Focusing on the Links between Splicing and Cancer in *C. elegans* Is Bright

Great strides have been taken in research to understand the involvement of splicing factors in biological processes such as cell proliferation, differentiation, migration, communication, and death (as discussed previoussly). RNAi screens have identified splicing factors that promote or inhibit cell proliferation. Transgenic assays have revealed cellular and subcellular localization of specific isoforms that may be involved in apoptotic or cell cycle regulation. Transparent cuticles and eggs make worms ideal specimens for the use of fluorescent reporters. Lineage tracing experiments have not only identified every cell in an adult worm but also precursors and cell behavior throughout development. Furthermore, only in a multicellular organism, such as *C. elegans*, can the effects of cell-to-cell signaling on processes such as differentiation or cell proliferation become apparent. High-throughput sequencing and bioinformatic analyses are opening a new chapter on comparisons in isoform usage between genetic backgrounds. The modEncode project seeks to identify functional elements within the *Drosophila* and *Caenorhabditis* genomes by providing access to high-quality gene expression datasets. Furthermore, the million-mutation project in *C. elegans* will allow researchers to obtain gene and even isoform-specific mutant strains for further study. Given the speed and depth of discoveries using methods such as RNAi in worms, this project should make *C. elegans* a core model to study alternative splicing and its link to genes homologous to those tied to cancer in humans.

## Figures and Tables

**Table 1:** Ras pathway components, regulators, interactors, and targets in *C. elegans*. Those marked with a √ produce 2 or more isoforms differing by at least 1 alternative exon (according to [4, 39]).

Ras pathway component/interactor			
ark-1		lin-10	
cdf-1	√	lin-25	
cnk-1		lin-31	
dab-1	√	lin-39	√
dpy-22		lin-45	√
dpy-23	√	lip-1	
egl-5	√	lrp-1	
egl-18		lst-1	√
egl-15	√	lst-2	
egl-17		lst-3	√
egl-19	√	lst-4	√
egl-30	√	mek-2	
elt-6		mpk-1/sur-1	√
eor-1		par-1	√
eor-2	√	ptp-2	
gap-1		rom-1	
gap-2	√	sem-4	
gpa-5		sem-5	
ksr-1		sli-1	√
ksr-2	√	soc-1	
let-23	√	sos-1/let-341	
let-60		sra-13	√
let-92		sur-2	
let-756		sur-5	
lin-1		sur-6	
lin-2	√	sur-7	√
lin-3	√	sur-8/soc-2	√
lin-7		unc-101	√
